# Supplementary material for: Impact of Acute Myeloid Leukemia Cells on the Metabolic Function of Bone Marrow Mesenchymal Stem Cells
Source: Int J Mol Sci. 2025 Aug 27;26(17):8301. doi: 10.3390/ijms26178301 (PMC12428039; doi:10.3390/ijms26178301)
Supplement: Supplementary file 1 [file ijms-26-08301-s001.zip › ijms-3610155-supplementary.pdf]

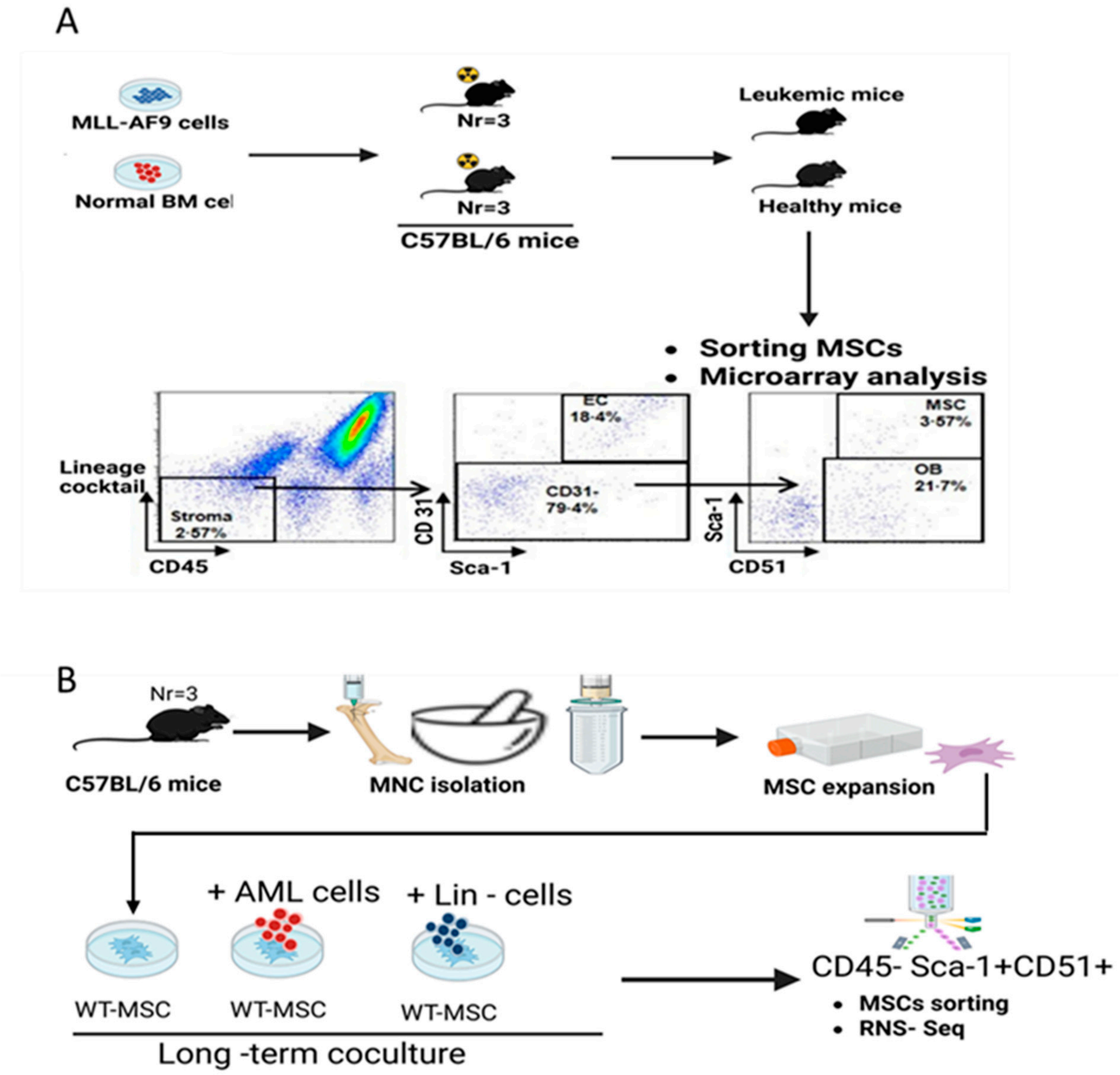

Figure S1. Molecular changes in MSCs cocultured with AML cells and in MSCs from leukemic and non-leukemic mice. (A) Schematic illustration of steps involved in the generation and sorting of MSCs. MLL–AF9 leukemic cells and healthy bone marrow cells were transplanted into sublethally irradiated recipient mice. The femora tibiae, and humeri were crushed with a mortar and pestle for the extraction of endosteal stromal elements by collagenase 1. The various cell types were analyzed for cell surface marker expression by flow cytometry. CD45−Lin−CD31−Sca-1+CD51+ MSCs were sorted and analyzed by RNA microarray analysis. (B) Schematic illustration of steps involved in the generation and sorting of MSCs in vitro. The femora tibiae, and humeri were crushed with a mortar and pestle for the extraction of endosteal stromal elements by collagenase 1 from healthy mice and bone marrow mononuclear cells were seeded in adherent flask to expand the MSCs in vitro. WT-MSCs coculture either alone or with AML cells and healthy lineage negative cells for long- term coculture. MSCs sorted (CD45−Sca-1+CD51+ MSCs) were sorted and analyzed by RNA-seq analysis.

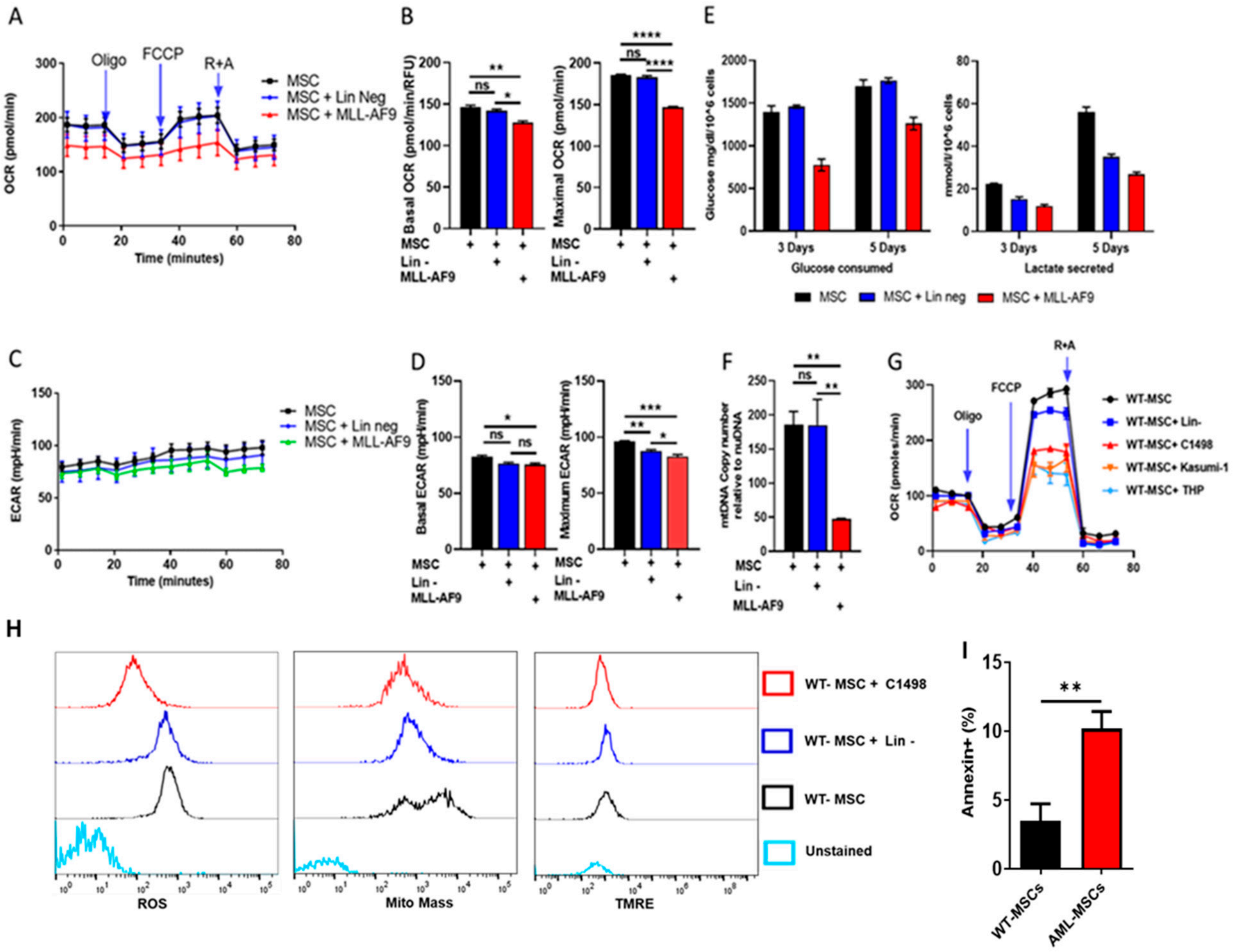

Figure S2. AML cells coculture with MSCs alter the cell metabolism. (A) Murine primary MSCs coculture with primary AML cells (MLL-AF9) for 5 days then sorted CD45-CD51+SCA-1+ MSCs subjected to Seahorse analyses to monitor oxygen consumption rate (OCR) as an assessment of oxidative phosphorylation. (B) Showed oxygen consumption rate (OCR) at basal level (left panel) and (right panel), Showed oxygen consumption rate (OCR) at maximal level. (C) Seahorse analyses to monitor extracellular acidification rate (ECAR) as an assessment of glycolysis. (D) showed extracellular acidification rate (ECAR) at basal level (left panel) and (right panel), Showed extracellular acidification rate (ECAR) at maximal level. (E) Culture for 3 and 5 days to measure the glucose consumed (left panel) and lactate secreted (right panel) by MSCs. (F) Mitochondrial DNA (mtDNA) copy number is calculated by real-time amplification of mtDNA and nuDNA genes as reported (37). The primers used in mtDNA measurement are listed in (Suppl. Table S2). (G) Murine MSCs cocultured with wild type Lin- cells, C1498, Kasumi and THP-1 cells for 5 days, thereafter (CD45-CD51+SCA-1+) MSCs were FACs sorted and Seahorse mito-stress analyses was performed to monitor OCR as an assessment of oxidative phosphorylation. (H) Flow cytometry gating and histogram measuring ROS, Mito Mass and membrane potential (TMRE). (I) Apoptosis based flowcytometry assay. The figure shows a representative data of three independent experiments in triplicates shown. The one ANOVA analysis was used to determine the levels of significance * $p \leq 0.05$, ** $p \leq 0.01$, *** $p \leq 0.005$ and **** $p \leq 0.001$.

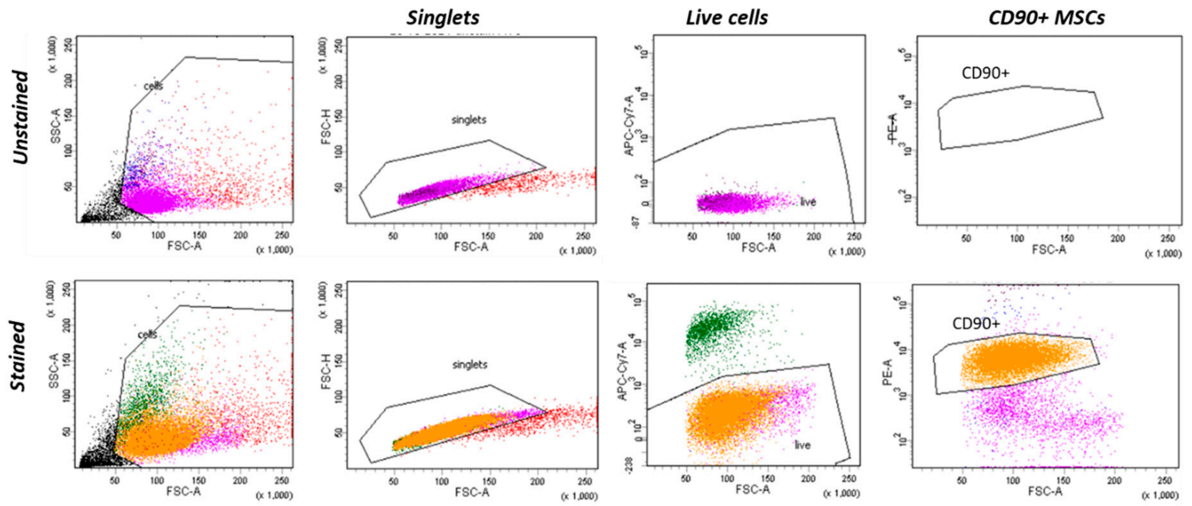

Figure S3. AML cells coculture with MSCs alter the cell metabolism. (A) HS-5 cells coculture with AML cells (MOLM-13) for 5 days then sorted MSCs CD90+ subjected to Seahorse and flowcytometry based analysis.

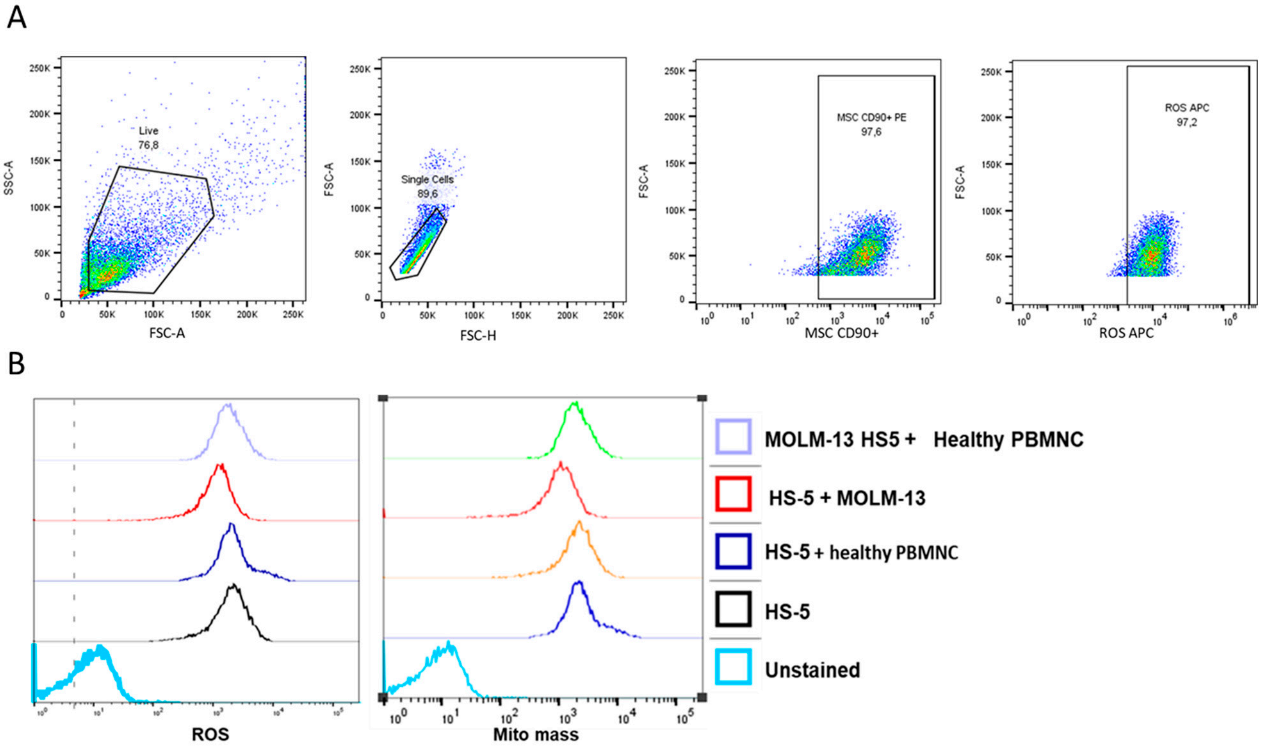

Figure S4. Metabolic profiling of MSCs Exposed to AML cells and reintroduced to healthy PBMCs impact on ROS and Mitochondrial Mass. (A) HS-5 cells were cultured alone or co-cultured with AML cells (MOLM-13) or healthy PBMCs for 5 days. After removing the AML cells, healthy PBMCs were added for 3 additional days. MSCs (CD90+) were then gated for flow cytometry analysis to measure ROS and mitochondrial mass. (B) Flow cytometry gating strategy and histograms illustrating the measurement of ROS (right panel) and mitochondrial mass (left panel).

Suppl. Table S1: Characteristics of patient samples used for MSCs immortalization and coculture assays.

MSCs	Diagnosis	Cytogenetics
AML-MSC	AML, FAB M3	(t15;17)(q22;q21)
HD-MSC	Normal BM purchased from LONZA (Cologne, Germany)	

AML=acute myeloid leukaemia, FAB= French American Britain classification

Supp. Table S2: mtDNA quantification primers.

mtDNA quantification primers	
Mouse actB F	CGGCTTGCGGGTGTTAAAAG
Mouse actB R	CGTGATCGTAGCGTCTGGTT
Human B2M F	TGCTGTCTCCATGTTTGATGTATCT
Human B2M R	TCTCTGCTCCCCACCTCTAAGT
Mouse Cyt B F	CTTCATGTCGGACGAGGCTTA
Mouse Cyt B R	TGTGGCTATGACTGCGAACA
humantRNALeu F	CACCCAAGAACAGGGTTTGT
humantRNALeu R	TGGCCATGGGTATGTTGTTA

Supp. Table S3:

Concentrations used in seahorse base media and Optimized inhibitor concentrations used in seahorse mito-stress and Glycolysis stress experiments.

	Glucose	L-Glutamine	Sodium Pyruvate
MSC	25 mM	4 mM	1 mM

Cell Type	Cells per well	Oligomycin	FCCP	Rotenone+ Antimycin	2-DG
MSC	20,000	2 μM	2 μM	500nM	100 mM
